# Nutritional risk factors and eating behaviors in adolescents with pectus excavatum: new approach by using cluster analysis

**DOI:** 10.1007/s00383-026-06305-w

**Published:** 2026-02-05

**Authors:** Sevde Kahraman, Yusuf Celik

**Affiliations:** 1 Faculty of Health Sciences, Department of Nutrition and Dietetics, Biruni University, Istanbul, Turkey; 2 Medical Faculty, Biostatistics Department, Biruni University, Istanbul, Turkey

**Keywords:** Pectus excavatum, Nutrition, Eating behavior, Breastfeeding, Cluster analysis

## Abstract

**Purpose:**

This study aimed to assess eating behaviors, dietary habits, and nutritional history in adolescents with pectus excavatum, and to examine the relationships between anthropometric, biochemical, and behavioral variables using multivariate cluster analysis.

**Methods:**

A total of 25 adolescents (21 males, 4 females) aged 11–18 years with a diagnosis of pectus excavatum participated in this cross-sectional study. Data were collected through sociodemographic surveys, anthropometric measurements, serum ferritin levels, and the Adolescent Eating Behavior Assessment Scale. Cluster analysis was applied to identify patterns between nutritional, anthropometric, biochemical, and behavioral factors.

**Results:**

Malnutrition was observed in 16% of participants, thinness in 20%, and short stature in 16%. Low serum ferritin levels were found in 20% of the cohort. Nutritional problems were reported by 72% of adolescents, mainly appetite loss (55.6%) and food neophobia (44.4%). Only 44% had been breastfed up to two years, and the majority (96%) exhibited moderate eating behaviors. Cluster analysis revealed two main clusters linking nutritional and behavioral variables with biochemical status and lifestyle factors.

**Conclusion:**

This study is the first to highlight the relationship between eating behaviors, breastfeeding duration, and daily meal frequency in adolescents with pectus excavatum. The findings emphasize the importance of early nutritional assessment and tailored interventions to support growth and overall health in this population.

## Introduction

Pectus excavatum is the most common congenital chest wall deformity and occurs approximately five times more frequently in males than females [1]. Embryologically, it arises from abnormal fusion between the ribs and the sternum, resulting in characteristic sternal depression [2]. While its physiological impact on cardiopulmonary function remains debated, many adolescents with pectus excavatum experience reduced exercise tolerance alongside significant psychosocial distress linked to body image dissatisfaction [1, 2].

Although much of the literature has emphasized anatomical, functional, and psychological domains, nutrition-related factors in pectus excavatum have been largely underexplored. A recent study by Kahrıman et al. (2025) demonstrated that nutritional education significantly improved anthropometric measures-including body weight, height-for-age, and body mass index (BMI)- and increased intake of energy, protein, and fat in adolescents with moderate pectus excavatum, suggesting that dietary interventions can positively influence both growth and nutritional status in this population [3].

Moreover, a broader pediatric study comparing adolescents with pectus excavatum and pectus carinatum to healthy controls found that those with chest wall deformities exhibited lower body weight, poorer physical function, and compromised psychosocial wellbeing. These findings imply that disordered eating behaviors or suboptimal nutritional intake may contribute to the physical and psychosocial challenges observed in this group [4]. Several studies have highlighted growth disturbances in patients with chest wall deformities. In a cross-sectional study conducted in Korea, Park et al. reported that the height, weight, and BMI of patients with pectus excavatum were significantly lower than those of age-matched healthy controls [5]. Similarly, in a cohort from Türkiye, Kuru et al. found that 32.5% of children with pectus excavatum and 26.4% of those with pectus carinatum were classified as underweight, further underscoring the nutritional and growth-related challenges associated with these deformities [6].

Although previous studies have primarily focused on the anatomical, functional, and psychosocial dimensions of pectus excavatum, the nutritional domain has remained largely underexplored. In particular, there is a notable ‘nutrition gap’ in the literature, as no studies to date have systematically evaluated the eating behaviors, dietary habits, or nutritional history of adolescents with pectus excavatum. Addressing this gap is essential, since nutritional status may influence growth, exercise tolerance, and even treatment outcomes in this patient group. The aim of this study was to evaluate the eating behaviors, dietary habits, and nutritional history of adolescents diagnosed with pectus excavatum, and to investigate the relationships between anthropometric, biochemical, and behavioral variables through multivariate cluster analysis.

## Materials and methods

This cross-sectional study aimed to assess the eating behaviors, dietary habits, nutritional history, and anthropometric measurements of adolescents aged 11–18 years diagnosed with pectus excavatum, and to investigate the relationships among anthropometric, biochemical, and behavioral variables using multivariate cluster analysis. The study was approved by the Clinical Research Ethics Committee of Biruni University on April 21, 2025 (approval number; 2024–BİAEK/09–10). Written informed consent was obtained from the parents prior to data collection, and verbal assent was obtained from all participating adolescent.

## Study population

This study included 25 children (21 males, 4 females) between the ages of 11 and 18 who were diagnosed with pectus excavatum and admitted to hospitals and private outpatient clinics in Istanbul between April 2025 and June 2025. The inclusion of 25 patients with the relatively rare condition of pectus excavatum aligns with methodological recommendations suggesting that exploratory studies should include sample sizes above 20 [7]. The inclusion criteria for the study were being diagnosed with pectus excavatum and being under 18 years of age. Participants who were over 18 years of age and had another disease diagnosis that could affect the respiratory system were excluded from the study.

All participants in this study had a clinical diagnosis of pectus excavatum, which was confirmed by either a pediatric or thoracic surgeon. However, detailed surgical parameters such as deformity severity indices (e.g., Haller index), cardiopulmonary functional assessments, the presence of syndromic associations (e.g., Marfan syndrome), and treatment status (surgical correction versus conservative follow-up) were not systematically available and therefore could not be included in the analysis. At the time of enrollment, none of the participants had undergone corrective surgery for pectus excavatum, and all were under routine clinical follow-up. This study was primarily designed to explore nutritional and behavioral characteristics rather than surgical severity or operative outcomes.

The following data and measurements were obtained from the children: a sociodemographic data form; anthropometric measurements (body weight, height, body mass index, waist circumference, hip circumference, upper middle arm circumference); and an Adolescent Eating Behavior Assessment scale. The serum ferritin levels of the children were documented according to the most recent blood test conducted within the preceding six months. For the interpretation of serum ferritin levels, the reference intervals provided by the laboratory that performed the analysis were used.

## Questionnaire on sociodemographic information

Data were collected through face-to-face interviews with volunteers who consented to participate, using a questionnaire that addressed demographic characteristics and dietary practices.

## Anthropometric measurements

Anthropometric measurements of weight, height, body mass index (BMI), mid-upper-arm circumference (MUAC), waist circumferences (WC), and hip circumferences (HC) measured. All measurements were performed by study staff who had been trained to use standardized methods. Weight was measured (in minimal clothes, with shoes and jackets removed) using calibrated electronic weighing scales (Phoenix PPS-160) and was recorded to the nearest 0.1 kg. Standing height was measured (shoes, hat removed) using a height stadiometer (Escala) and was recorded to the nearest 0.1 cm. BMI was calculated as BMI = weight (in kg) ÷ height (in m^2^). MUAC was determined with a non-stretchable measuring tape and was recorded to the nearest millimeter.

WC and HC were measured using a semi-flexible tape measure, 2 cm above the iliac crest for waist and at the level of the widest circumference over the greater trochanters for hip [8].

We expressed these measures as sex–age-specific Z-scores and percentiles based on the WHO Child Growth Standards for weight-for-height, weight-for-age, BMI-for-age, and height-for-age [9]. Malnutrition was defined as a BMI z-score < − 3 standard deviations (SD), thinness as z-score between − 3 SD and − 2 SD, normal weight as z-score between − 2 SD and + 1 SD, overweight as z-score > 1 SD, and obesity as z-score > + 2 SD [10].

### Adolescent eating behavior assessment scale

The Adolescent Eating Behavior Assessment Scale was developed by Yahya Özdoğan in 2013 and designed as a metric scale. In this metric scale, responses were scored by placing a mark on the response axis and then measuring with a ruler, starting from the left for positive items and from the right for negative items. The maximum possible score on the scale is 580, while the minimum score is 0. Higher scores indicate positive behavior and better nutritional knowledge. The evaluation criteria of the eating behavior scale were defined as follows: ≤145 points = poor, 146–290 points = moderate, 291–435 points = good, and ≥ 436 points = very good. To assess the reliability of the scale, the Cronbach’s Alpha method was employed. Following the factor analysis of the data obtained from the application of the scale, the Cronbach’s Alpha coefficient was found to be 0.84 [11]. This scale has been used with adolescent populations to evaluate eating behavior patterns, nutrition-related awareness, and to identify individuals at risk of suboptimal dietary behaviors. In clinical and research settings, it provides a behavioral framework to support nutritional assessment and guide targeted nutritional counselling.

## Ethical consideration

The study was approved by the Clinical Research Ethics Committee of Biruni University on April 21, 2025 (approval number; 2024–BİAEK/09–10). Written informed consent was obtained from the parents prior to data collection, and verbal assent was obtained from all participating children.

## Statistical analysis

Mean, standard deviation (X̄±SD) and frequency and percent (%) values were calculated for continuous and discrete variables respectively.

Cluster analysis of multivariate statistical method was used to find the dendrogram of variables. This analysis involves clustering a set of objects such that all objects within a cluster are similar to one another, while simultaneously ensuring that they are distinctly different from objects in other clusters. It is a key part of exploratory data analysis, whereby observations are divided into meaningful groups whose members share common characteristics. This technique is widely used in statistical data analysis and in many fields, including pattern recognition, image analysis, information retrieval, bioinformatics, data compression, computer graphics and machine learning [12].

Normality of variables was analyzed with the Kolmogorov-Smirnov (KS) test. The hypotheses were two-sided, and *p* ≤ 0.05 was considered to be a statistically significant difference, and all statistical analyses were evaluated using R software/programming (version 3.6.2 (2019-12-12) - CRAN).

## Results

The research was conducted on 25 adolescents (21 males and 4 females) aged between 11 and 18 years (mean age: 15.0 ± 3.0 years) who were diagnosed with pectus excavatum in Istanbul, Türkiye. Tables 1 and 2 present descriptive sociodemographic characteristics and anthropometric measurements of the participants. According to BMI-for-age Z-scores, 20% of the participants had thinness, while 16% had malnutrition. Additionally, according to height-for-age Z-scores, 16% were identified as having short stature. Low serum ferritin levels were observed in 20% of the adolescents. Nutritional problems were present in 72% of the cohort, most commonly loss of appetite (55.6%) and food neophobia (44.4%). Only 44% of participants had been breastfed up to two years of age. Based on the Adolescent Eating Behavior Assessment Scale, the majority (96%) were classified as having moderate eating behaviors, while only 4% demonstrated good eating behaviors.

**Table 1:** Descriptive statistics: frequency distributions of children and parents

	*n* (%)
Sex	
Female	4 (16.0)
Male	21 (84.0)
Height for age Z-score classification	
Normal	21 (84.0)
Short (malnutrition)	4 (16.0)
BMI for age Z-score classification	
Normal	16 (64.0)
Thinness	5 (20.0)
Malnutrition	4 (16.0)
Observed nutritional problems	
Yes	18 (72.0)
No	7 (28.0)
Causes nutritional problems	
Loss of appetite	10 (55.6)
Neophobia	8 (44.4)
Eating patterns	
Self	25 (100.0)
Observed awareness of healthy nutrition	
No	11 (44.0)
Partially	14 (56.0)
Duration of breastfeeding	
0–6 months	5 (20.0)
7–12 months	9 (36.0)
13–24 months	11 (44.0)
Breakfast consumption	
Yes	1 (4.0)
No	11 (44.0)
Irregular	13 (52.0)
Symptoms after consuming packaged foods	
Hyperactivity	1 (4.0)
Inattention	3 (12.0)
Headache	11 (44.0)
Allergy and skin problems	10 (40.0)
Serum ferritin status classification	
Low	5 (20.0)
Normal	20 (80.0)
Classification of Adolescent Eating Behavior Assessment Scale	
Bad	0 (0.0)
Moderate	24 (96.0)
Good	1 (4.0)
Very good	0 (0.0)
Parental meal skipping behavior	
Yes	24 (96.0)
No	1 (4.0)
Maternal tobacco use during pregnancy	
No	25 (100.0)

**Table 2:** Descriptive statistics: mean values for children

	Man (*n* = 21) Mean ± SD	Woman (*n* = 4) Mean ± SD	Total (*n* = 25) Mean ± SD
Age (year)	15.4 ± 2.7	13.0 ± 4.0	15.0 ± 3.0
Weight (kg)	46.4 ± 11.5	34.3 ± 14.4	44.0 ± 12.7
Height (cm)	165.9 ± 13.6	142.5 ± 16.6	161.2 ± 16.8
BMI (kg/m^2^)	16.6 ± 2.1	16.2 ± 2.8	16.5 ± 2.2
Waist circumference (cm)	67.9 ± 7.8	60.8 ± 12.1	66.5 ± 8.9
Hip circumference (cm)	82.1 ± 10.8	77.0 ± 16.1	81.1 ± 11.7
MUAC (cm)	22.6 ± 2.4	19.3 ± 3.2	21.9 ± 2.9
Sleep duration (h/d)	8.2 ± 0.7	8.8 ± 0.5	8.3 ± 0.7
Water consumption (mL/d)	1618.8 ± 310.3	1350 ± 173.2	1565.0 ± 304.8
Number of daily meal	3.0 ± 0.5	2.8 ± 0.5	3.0 ± 0.5

BMI: body mass index; MUAC: mid-upper arm circumference; SD: standard deviation

**Fig. 1:**
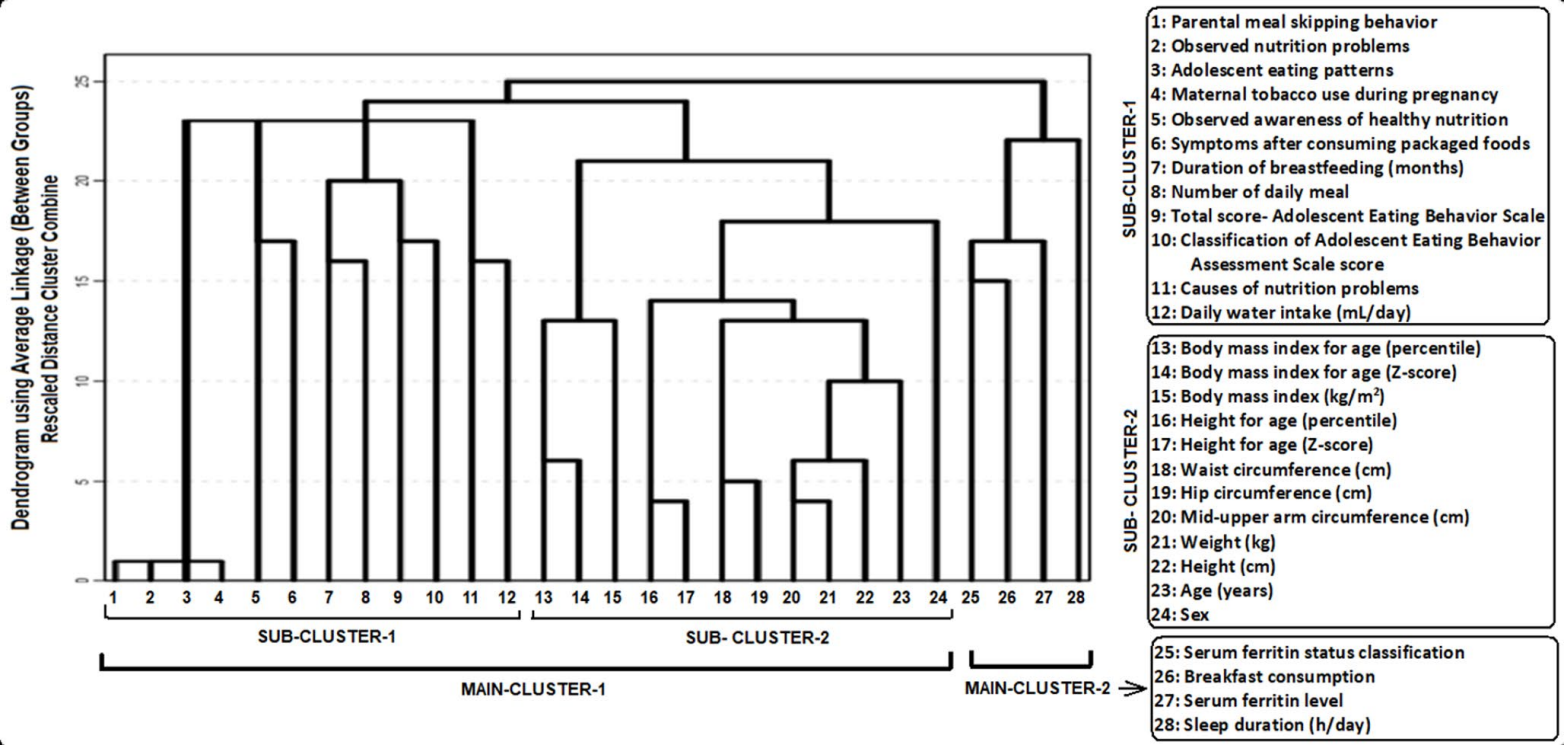
Dendrogram showing variable clusters and the relationships between them, found with the “Hierarchical Clustering Method”

The results of the hierarchical cluster analysis are shown in Fig. 1, illustrating the interrelationships among anthropometric, biochemical, and behavioral variables. Hierarchical cluster analysis evaluates all variables simultaneously to identify close associations, and the use of a dendrogram allows for a clearer visualization of these relationships compared with other methods, as it prevents the loss of information.

Cluster analysis, which analyzes all variables together, has created a subset and a main cluster according to which variables have a strong relationship with each other. When Fig. 1 is examined, it is seen that the variables are formed by two strong subsets (which are also formed by subsets) and two main clusters. These are as follows, respectively.

### Main cluster-I

#### Sub-cluster-I (SC-I)

1. Parental meal skipping behavior.
2. Observed nutrition problems.
3. Adolescent eating patterns.
4. Maternal tobacco use during pregnancy.
5. Observed awareness of healthy nutrition.
6. Symptoms after consuming packaged foods.
7. Duration of breastfeeding (months).
8. Number of daily meal.
9. Total score-Adolescent Eating Behavior Scale.
10. Classification of Adolescent Eating Behavior Scale score.
11. Causes of nutrition problems
12. Daily water intake (mL/day)

### Sub-cluster-II (SC-II

13. Body mass index for age (percentile)
14. Body mass index for age (Z-score)
15. Body mass index (kg/m2)16. Height for age (percentile)
16. Height for age (Z-score)
17. Waist circumference (cm)
18. Hip circumference (cm)
19. Mid-upper arm circumference (cm)
20. Weight (kg)
21. Height (cm)
22. Age (years)
23. Sex

### Main cluster-II

24. Serum ferritin status classification
25. Breakfast consumption
26. Serum ferritin level
27. Sleep duration (h/day)

The hierarchical cluster analysis identified groups of variables that clustered together based on similarity patterns. These clusters represent co-occurrence of variables rather than causal or clinically deterministic relationships. Main Cluster I comprised two sub-clusters. Sub-cluster I included variables related to eating behaviors, parental and maternal factors, dietary awareness, and daily habits, while Sub-cluster II grouped anthropometric and demographic characteristics. Main Cluster II consisted of biochemical and lifestyle-related variables, including serum ferritin status, breakfast consumption, and sleep duration. These findings illustrate patterns of variable similarity within the study population.

## Discussion

The study included 25 patients, the majority of whom (84.0%) were male. This predominance aligns with previous reports indicating that pectus deformities occur more frequently in males [1, 13].

In the study conducted by Park et al., the anthropometric measurements of 1,371 patients diagnosed with pectus excavatum were evaluated. The body weight, height, and body mass index values of these patients were found to be significantly lower than those of the healthy population prior to thoracic surgery (*p* < 0.001) [5]. Rebeis et al. (2013) reported a statistically significant difference in anthropometric index values among patients with pectus excavatum aged 11–40 years, noting that female participants had lower index values than their male counterparts [14]. In a study from Türkiye, 32.5% of children with pectus excavatum and 26.4% of those with pectus carinatum were reported to be underweight [6]. According to another study from Türkiye, 65% of individuals with pectus excavatum were classified as malnourished, while 35% exhibited normal nutritional status [3]. These results highlight the susceptibility of individuals with pectus deformities to malnutrition. As noted in previous studies, inadequate nutritional status may negatively influence disease progression and elevate postoperative risks in patients requiring surgical intervention [15]. In our study, age-specific BMI Z-scores were assessed in adolescents with pectus excavatum. Based on these findings, 20% of the participants were classified as having thinness and 16% as having malnutrition, while 16% were also identified as having short stature according to height-for-age.

Pectus excavatum is considered the most common congenital deformity of the chest wall, and its underlying cause is still unknown. Patients with pectus excavatum may present with exercise intolerance, cardiopulmonary limitations, musculoskeletal changes, and psychosocial distress, the latter often exceeding the physical impairment [16, 17]. Malnutrition in these patients may develop as a result of underlying health problems [3, 5, 6, 14]; nevertheless, other nutritional issues have not been previously documented. In our study, most adolescents with pectus excavatum (72%) were found to have nutritional problems, with loss of appetite (55.6%) and food neophobia (44.4%) being the main contributing factors.

According to a cross-sectional study by Monyeki et al. (2024), poor iron status in South African adolescents-reflected by low haemoglobin and ferritin levels-was significantly associated with reduced lower-body explosive power and diminished cardiorespiratory fitness, underscoring the detrimental impact of iron deficiency on exercise capacity [18]. In line with these findings, in our study, 20% of adolescents diagnosed with pectus excavatum were found to have low serum ferritin levels, suggesting that iron deficiency may further contribute to exercise intolerance in this patient group.

Although infants are recommended to be breastfed until the age of two [19–21], our study revealed that only 44% of adolescents with pectus excavatum had received breastfeeding for this duration.

The cross-sectional study by Zięba et al. (2025) highlights that adolescents’ nutritional knowledge, emotional regulation, and media influence are significant determinants of their eating behaviors, with a notable proportion exhibiting susceptibility to misinformation and emotional eating triggers [22]. Similarly, the systematic review by Maneschy et al. (2024) emphasized that ‘food approach’ behaviors in children and adolescents are often associated with higher intake of both energy-dense foods and fruits and vegetables, whereas ‘food avoidant’ behaviors tend to reduce overall intake but increase snacking frequency. Taken together, these findings illustrate how adolescent eating behaviors are shaped by a complex interplay of psychosocial and environmental factors [23]. In line with this evidence, in our study of adolescents with pectus excavatum, 96% were categorized as having ‘moderate’ eating behaviors, while only 4% achieved a ‘good’ classification. Although there is no study in the literature assessing the eating behaviors of patients diagnosed with pectus excavatum, understanding such behaviors may provide valuable insights for clinical management and nutritional interventions.

An important limitation of this study is the lack of detailed surgical characterization of pectus excavatum severity. Parameters such as the Haller index, cardiopulmonary functional measures, associated syndromes, and treatment status were unavailable; therefore, the findings cannot be directly linked to deformity severity or surgical decision-making. Accordingly, the nutritional and behavioral patterns identified in this study should be interpreted as descriptive and hypothesis-generating rather than predictive of surgical outcomes. In addition, several potential confounding factors (including pubertal stage, psychological factors (such as body image perception and anxiety), physical activity levels, and socioeconomic background) were not assessed. These factors are known to independently influence dietary behaviors, appetite regulation, and growth during adolescence. Moreover, information regarding iron supplementation or prior anemia history was not systematically collected, which may have influenced serum ferritin levels. Another limitation relates to iron status assessment. Serum ferritin values were obtained from the most recent laboratory records within the preceding six months, which may have introduced temporal variability. Furthermore, hemoglobin levels, inflammatory markers (e.g., C-reactive protein), and standardized laboratory data were not consistently available. As ferritin is an acute-phase reactant, these limitations restrict the ability to distinguish true iron deficiency from inflammation-related changes. Therefore, ferritin findings should be interpreted with caution. Future studies incorporating standardized surgical, nutritional, and laboratory assessments are needed to strengthen clinical interpretation.

## Conclusion

This study is the first to evaluate eating behaviors, breastfeeding duration, and daily meal frequency in adolescents with pectus excavatum. Our findings revealed that nutritional problems were highly prevalent in this group, with 20% showing thinness, 16% showing malnutrition, 20% low serum ferritin levels, and 72% reporting issues such as loss of appetite and food neophobia. Moreover, only 44% of the participants had been breastfed up to two years, and the vast majority (96%) demonstrated moderate rather than good eating behaviors. Taken together, these results suggest that, beyond anatomical and psychosocial aspects, adolescents with pectus excavatum are at considerable nutritional risk. Early dietary assessment and individualized nutritional interventions should therefore be integrated into the management of these patients to support growth, improve health outcomes, and potentially enhance treatment success.

## Data Availability

The data supporting the findings of this study are not publicly available due to ethical restrictions and the inclusion of identifiable patient information but are available from the corresponding author upon reasonable request.
